# Ranking Rule-Based Automatic Explanations for Machine Learning Predictions on Asthma Hospital Encounters in Patients With Asthma: Retrospective Cohort Study

**DOI:** 10.2196/28287

**Published:** 2021-08-11

**Authors:** Xiaoyi Zhang, Gang Luo

**Affiliations:** 1 Department of Biomedical Informatics and Medical Education University of Washington Seattle, WA United States

**Keywords:** asthma, clinical decision support, machine learning, patient care management, forecasting

## Abstract

**Background:**

Asthma hospital encounters impose a heavy burden on the health care system. To improve preventive care and outcomes for patients with asthma, we recently developed a black-box machine learning model to predict whether a patient with asthma will have one or more asthma hospital encounters in the succeeding 12 months. Our model is more accurate than previous models. However, black-box machine learning models do not explain their predictions, which forms a barrier to widespread clinical adoption. To solve this issue, we previously developed a method to automatically provide rule-based explanations for the model’s predictions and to suggest tailored interventions without sacrificing model performance. For an average patient correctly predicted by our model to have future asthma hospital encounters, our explanation method generated over 5000 rule-based explanations, if any. However, the user of the automated explanation function, often a busy clinician, will want to quickly obtain the most useful information for a patient by viewing only the top few explanations. Therefore, a methodology is required to appropriately rank the explanations generated for a patient. However, this is currently an open problem.

**Objective:**

The aim of this study is to develop a method to appropriately rank the rule-based explanations that our automated explanation method generates for a patient.

**Methods:**

We developed a ranking method that struck a balance among multiple factors. Through a secondary analysis of 82,888 data instances of adults with asthma from the University of Washington Medicine between 2011 and 2018, we demonstrated our ranking method on the test case of predicting asthma hospital encounters in patients with asthma.

**Results:**

For each patient predicted to have asthma hospital encounters in the succeeding 12 months, the top few explanations returned by our ranking method typically have high quality and low redundancy. Many top-ranked explanations provide useful insights on the various aspects of the patient’s situation, which cannot be easily obtained by viewing the patient’s data in the current electronic health record system.

**Conclusions:**

The explanation ranking module is an essential component of the automated explanation function, and it addresses the interpretability issue that deters the widespread adoption of machine learning predictive models in clinical practice. In the next few years, we plan to test our explanation ranking method on predictive modeling problems addressing other diseases as well as on data from other health care systems.

**International Registered Report Identifier (IRRID):**

RR2-10.2196/5039

## Introduction

### Background

Approximately 7.7% of Americans and over 339 million people worldwide have asthma [1,2]. Asthma incurs a total medical cost of US $50 billion [3], 1,564,440 emergency department (ED) visits, and 182,620 inpatient stays annually in the United States [1]. A primary goal of asthma management is to decrease the number of asthma hospital encounters, namely, ED visits and inpatient stays. The state-of-the-art approach for achieving this goal is to deploy a predictive model to identify patients at high risk of having poor outcomes in the future. Once identified, the patient is placed into a care management program. The program will assign a care manager to regularly contact the patient to assess asthma control status, adjust asthma medications when needed, and help schedule appointments for health and other relevant services. Many health plans, including those in 9 of 12 metropolitan communities [4], and many health care systems, such as the University of Washington Medicine (UWM), Intermountain Healthcare, and Kaiser Permanente Northern California, currently use this approach [5]. When used correctly, this approach prevents up to 40% of future asthma hospital encounters [4,6-9].

Due to limited capacity, a care management program can serve at most 3% of patients [10]. To maximize the effectiveness of these programs, an accurate predictive model should be used to identify the highest-risk patients. For this purpose, we recently developed a machine learning model powered by extreme gradient boosting (XGBoost) [11] on UWM data to predict which patients with asthma will have asthma hospital encounters in the succeeding 12 months [12]. Compared with previous models [5,13-26], this model is more accurate and improves the area under the receiver operating characteristic curve by ≥0.09. In addition, we previously developed a method to automatically explain the model’s predictions in the form of rules and to suggest tailored interventions without sacrificing model performance [27,28]. Our method works for any black-box machine learning predictive model built on tabular data and addresses the interpretability issue that deters the widespread adoption of machine learning predictive models in clinical practice. Among all the published automated explanation methods for machine learning predictions [29,30], only our method can automatically recommend tailored interventions. For an average patient whom our UWM model correctly predicted to have future asthma hospital encounters, our method generated over 5000 rule-based explanations, if any [27]. The amount of nonredundant information in these explanations is usually two orders of magnitude less than the number of explanations, as multiple explanations often share some common components. The user of the automatic explanation function wants to quickly obtain the most useful information for a patient by viewing only the top few explanations. Therefore, we need to appropriately rank the explanations generated for each patient. Currently an open problem, procedures for appropriately ranking explanations are particularly important for the adoption of our automated explanation method in a busy clinical environment.

### Objectives

To fill this gap, the aim of this study is to develop a method to appropriately rank the rule-based explanations generated by our automated explanation method [27,28] for a patient. We demonstrated our explanation ranking method in a test case that predicts asthma hospital encounters in patients with asthma.

## Methods

### Items Reused From Our Previous Papers

We reused the following items from our previous papers [12,27]: patient cohort, prediction target (ie, the dependent variable), features (ie, independent variables), data set, data preprocessing method, predictive model, cutoff threshold for binary classification, and automated explanation method. A list of symbols used in this paper is provided in Textbox 1.

**List of symbols.**

**List of Symbols**

- *C_r_*: confidence of the association rule *r*
- *d*: decay constant
- *f*(*d, p_i_, r*): exponential decay function computed for the feature-value pair item *p_i_* on the left-hand side of the association rule *r*
- *f*: feature
- *m*: number of feature-value pair items on the left-hand side of an association rule
- max(*v_r_*(x)): maximum value of the variable *v_r_*(x) across all the rules found for the patient
- mean(*f*(*r*)): mean of *f*(*d, p_i_, r*) over all the feature-value pair items on the left-hand side of the association rule *r*
- min(*v_r_*(x)): minimum value of the variable *v_r_*(x) across all the rules found for the patient
- *n*: maximum number of top-ranked explanations that are allowed to be displayed initially
- norm(): normalization function
- *N_r_*: number of feature-value pair items on the left-hand side of the association rule *r*
- *p*: feature-value pair item
- *p_i_*: the *i*-th feature-value pair item on the left-hand side of an association rule
- *q*: number of association rules generated by our automated explanation method for the patient
- *r*: association rule
- *score_p_*: ranking score of the feature-value pair item *p*
- *score_r_*: ranking score of the association rule *r*
- *S_r_*: commonality of the association rule *r*
- *t, t_i_*: number of times that a feature-value pair item appears in the higher-ranked rules
- *u*: a value or a range
- *v*: outcome value
- *v_r_*(x): variable whose value on the association rule *r* is *x*
- *w_a_*: weight for the term *δ_actionable_*(*r*) in the rule scoring function
- *w_b_*: weight for the term *δ_actionable_*(*p*) in the item scoring function
- *w_c_*: weight for the term norm(*C_r_*) in the rule scoring function
- *w_d_*: weight for the term mean(*f*(*r*)) in the rule scoring function
- *w_g_*: weight for the term exp(−*d·t*) in the item scoring function
- *w_n_*: weight for the term norm(*N_r_*) in the rule scoring function
- *w_s_*: weight for the term norm(log_10_*S_r_*) in the rule scoring function
- *x*: value
- *δ_actionable_*(*p*): indicator function for whether the feature-value pair item *p* is actionable
- *δ_actionable_*(*r*): indicator function for whether the association rule *r* is actionable

### Ethics Approval

The institutional review board of the UWM approved this secondary analysis retrospective cohort study.

### Patient Cohort

In Washington State, the UWM is the largest academic health care system. Its enterprise data warehouse stores clinical and administrative data from 3 hospitals and 12 clinics for adults. The patient cohort included all adult patients with asthma (aged ≥18 years) who received care at any of these UWM facilities between 2011 and 2018. In a specific year, a patient was considered asthmatic if the patient had one or more asthma diagnosis codes (International Classification of Diseases [ICD], Tenth Revision: J45.x; ICD, Ninth Revision: 493.0x, 493.1x, 493.8x, 493.9x) documented in the encounter billing database during the year [13,31,32]. We excluded the patients who died during that year.

### Prediction Target

Given a patient deemed asthmatic in an index year, we wanted to predict whether the patient would experience any asthma hospital encounter at the UWM in the succeeding 12 months, that is, any ED visit or inpatient stay at the UWM with asthma (ICD-10: J45.x; ICD-9: 493.0x, 493.1x, 493.8x, 493.9x) as its principal diagnosis. In predictive model training and testing, the patient’s outcome in the succeeding 12 months was predicted using the patient’s data until the end of the year.

### Data Set

We used a structured administrative and clinical data set retrieved from the UWM’s enterprise data warehouse. This data set contained information recorded for the visits by the patient cohort to the 12 clinics and 3 hospitals of the UWM over the 9-year span of 2011-2019. As the prediction target was for the following 12 months, the effective data in the data set spanned across the 8-year period of 2011-2018.

### The Training and Test Set Split

We used the data from 2011 to 2017 as the training set to train the predictive model and to mine the association rules used by our automated explanation method. We used the data of 2018 as the test set to demonstrate our ranking method for the rule-based explanations generated by our automated explanation method.

### Predictive Model and Features

Our UWM model used the XGBoost classification algorithm [11] and 71 features to predict the prediction target. As our UWM model was built on a single computer whose memory could hold the entire data set, the exact greedy algorithm was used to find the best split for tree learning in XGBoost [11]. These 71 features are listed in Table S2 in Multimedia Appendix 1 of our previous paper [12]. They were constructed based on the structured attributes in our data set and described various aspects of the patient’s situation, such as demographics, encounters, diagnoses, laboratory tests, procedures, vital signs, and medications. An example feature is the patient’s mean length of stay for an ED visit in the past year. Every input data instance to our predictive model includes these 71 features. Features that are the same as or similar to these 71 features were formerly used to predict asthma hospital encounters in patients with asthma and to provide automatic explanations on Intermountain Healthcare data as well as on Kaiser Permanente Southern California data [28,33-35]. For binary classification, we set the cutoff threshold at the top 10% of patients predicted to be at the highest risk. Our previous study [12] showed that on the test set, our model reached an area under the receiver operating characteristic curve of 0.902, an accuracy of 90.6% (13,268/14,644), a sensitivity of 70.2% (153/218), a specificity of 90.91% (13,115/14,426), a positive predictive value of 10.45% (153/1464), and a negative predictive value of 99.51% (13,115/13,180).

### Review of Our Automated Explanation Method

#### Success Stories

Our automated explanation method [27,28] was designed as a general method that works for any machine learning predictive model built on tabular data. We initially demonstrated our method for predicting the diagnosis of type 2 diabetes [36]. Later, we successfully applied our method to predict asthma hospital encounters in patients with asthma on Intermountain Healthcare data [28], UWM data [27], and Kaiser Permanente Southern California data [34]. Other researchers have also successfully applied our method to project lung transplantation or death in patients with cystic fibrosis [37]; to project cardiac death in patients with cancer; and to use projections to manage heart transplant waiting list, posttransplant follow-ups, and preventive care in patients with cardiovascular diseases [38].

#### Main Idea

**Figure 1:**
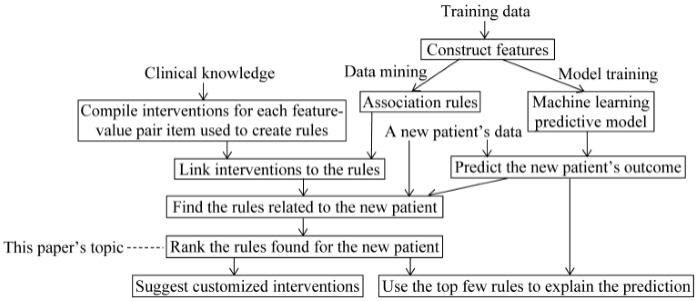
The flow diagram of our automated explanation method coupled with our explanation ranking method.

Our automated explanation method [27,28] uses class-based association rules [39,40] mined from historical data to explain a model’s predictions and to recommend tailored interventions. As shown in Figure 1, the association rules are constructed separately from the predictive model and are used solely to provide explanations rather than to make predictions. Thus, our automated explanation method can work with any machine learning predictive model built on tabular data with no performance penalty. That is, our method falls into the category of model-agnostic explanation methods, which are widely used to automatically explain machine learning predictions [29,30].

Before rule mining starts, an automated discretizing method based on the minimum description length principle [40,41] is first applied to the training set to convert continuous features into categorical features. The association rules are then mined from the training set using a standard method, such as Apriori [39]. Each rule shows that a feature pattern is linked to an outcome value and has the form

*p_1_* AND *p_2_* AND ...AND *p_m_ → v* **(1)**

Here, each item *p_i_* (1≤*i*≤*m*) is a feature-value pair (*f*, *u*). *u* is either the specific value of feature *f* or a range in which the value of *f* falls. For binary classification of a good versus a poor outcome, *v* is the poor outcome value; for example, the patient will have ≥1 inpatient stay or ED visit for asthma in the succeeding 12 months. For a patient fulfilling all of *p_1_*, *p_2_*, ..., and *p_m_*, the rule indicates that the patient’s outcome is likely to be *v*. An example rule is given below:

The patient had ≥13 ED visits in the past year AND the patient had ≥4 systemic corticosteroid prescriptions in the past year → The patient will likely have ≥1 inpatient stay or ED visit for asthma in the succeeding 12 months.

#### Constraints Put on the Association Rules

Our automated explanation method imposes several constraints on the association rules used by it. In this section, we review some of the constraints that are relevant to our explanation ranking method. For an association rule

*p_1_* AND *p_2_* AND ...AND *p_m_ → v*, **(2)**

*commonality* measures its coverage in the context of *v*; among all of the data instances linking to *v*, commonality is the percentage of data instances fulfilling *p_1_*, *p_2_*, ..., and *p_m_*. Meanwhile, *confidence* measures its precision; among all of the data instances fulfilling *p_1_*, *p_2_*, ..., and *p_m_*, the confidence is the percentage of data instances linking to *v*. For every association rule used by our automated explanation method, we require its commonality to be greater than or equal to a given minimum commonality threshold, such as 1%; its confidence to be greater than or equal to a given minimum confidence threshold, such as 50%; and its left-hand side to have no more than a given number (eg, 5) of feature-value pair items. As detailed in our previous papers [27,28], by setting the thresholds to these values, we can fulfill three goals concurrently. First, explanations can be given to most patients whom our UWM model correctly predicts as having ≥1 asthma hospital encounter in the succeeding 12 months. Second, the rule has sufficiently high confidence for the user of the automated explanation function to trust the rule. Third, no rule is overly complex.

#### The Explanation Method

For each feature-value pair item used to create association rules, a clinician in the development team of the automated explanation function precompiles 0 or more interventions. An item linking to at least one intervention is called actionable. The interventions related to the actionable items on the left-hand side of a rule are automatically linked to that rule. A rule linking to at least one intervention is called actionable.

For each patient predicted to have a poor outcome by the predictive model, the prediction is explained by the related association rules. For each such rule, the patient satisfies all of the feature-value pair items on its left-hand side. The poor outcome value appears on its right-hand side. Each rule delineates a reason for the patient’s predicted poor outcome. Every actionable rule is displayed along with its linked interventions. The user of the automated explanation function can choose from these tailored interventions for the patient. The rules mined from the training set typically cover common reasons for having poor outcomes. Nonetheless, some patients could have poor outcomes due to rare reasons, such as the patient was prescribed between three and seven asthma medications during the past year AND the patient was prescribed ≥11 distinct medications during the past year AND the patient has some drug or material allergy AND the patient had ≥1 active problem in the problem list during the past year. Hence, our explanation method usually explains the predictions for most, though not all, of the patients correctly predicted by the model to have poor outcomes.

### Ranking the Rule-Based Explanations Generated by Our Automated Explanation Method

#### Overview

For an average patient whom the predictive model predicts to have a poor outcome, our automated explanation method finds many related association rules, if any. Multiple rules often share some common feature-value pair items on their left-hand sides. To avoid overwhelming the user of the automated explanation function and to enable the user to quickly obtain the most useful information by viewing only the top few rules, we need to appropriately rank the rules found for a patient. As a rule often has a long description, a standard computer screen can show only a few rules simultaneously. To reduce the burden on the user, we present the rules in a manner similar to how a web search engine presents its search results for a keyword query. We chose a small number *n*, such as 3. The user can opt to change the value of *n*, for example, based on the size of the computer screen. If ≤*n* rules are found for the patient, we display all of these rules. Otherwise, if >*n* rules are found for the patient, we display the top *n* rules by default. If desired, the user can request to see more rules, for example, by dragging a vertical scroll bar or by clicking the *next page* button.

The main idea of our association rule ranking method is to consider multiple factors in the ranking process. The procedure incorporates these factors into a rule scoring function that strikes a balance among them and then ranks the rules found for a patient based on the scores computed for the rules in an iterative manner. In each iteration, the scores of the remaining rules are recomputed, and then, a rule is chosen from them. In the following, we describe our rule ranking method in detail.

#### Factors Considered in the Association Rule Ranking Process

When ranking the association rules found for a patient, we consider five factors:

1. *Factor 1*: All else being equal, a rule with a higher confidence is more precise and should rank higher.
2. *Factor 2*: All else being equal, a rule with a higher commonality covers a larger portion of patients with poor outcomes and should rank higher.
3. *Factor 3*: All else being equal, a rule with fewer feature-value pair items on its left-hand side is easier to comprehend and should rank higher.
4. *Factor 4*: In information retrieval, search engine users want to see diversified search results [42-44]. Similarly, the user of the automated explanation function wants to see diversified information in the top-ranked rules. Hence, all else being equal, a rule whose left-hand side has more items appearing in the higher-ranked rules should rank lower. The more times the items on the left-hand side of this rule appear in those rules, the lower this rule should rank.
5. *Factor 5*: The user of the automated explanation function wants to find suitable interventions for the patient. Thus, all else being equal, an actionable rule should rank higher than a nonactionable rule.

#### The Rule Scoring Function

We incorporate the five factors listed above into a rule scoring function to strike a balance among them. For an association rule

*r: p_1_* AND *p_2_* AND ...AND *p_m_ → v*, **(3)**

its ranking score is a linear combination of five terms, one per factor:

*score_r_=w_c_·*norm(*C_r_*)+*w_s_·*norm(log_10_*S_r_*)*−w_n_·*norm(*N_r_*)*+ w_d_·*mean(*f*(*r*))*+w_a_·*δ*_actionable_*(*r*) **(4)**

At a high level,

1. *C_r_* denotes *r*’s confidence. The term norm(*C_r_*) has a weight *w_c_*>0 and addresses factor 1.
2. *S_r_* denotes *r*’s commonality. The term norm(log_10_*S_r_*) has a weight *w_s_*>0 and addresses factor 2.
3. *N_r_* denotes the number of feature-value pair items on *r*’s left-hand side. The term norm(*N_r_*) has a weight *w_n_*>0 and addresses factor 3.
4. The term mean(*f*(*r*)) 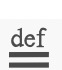 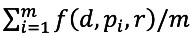 has a weight *w_d_*>0 and addresses factor 4. For each *i* (1≤*i*≤*m*), the function *f*(*d, p_i_, r*) is computed based on the number of times the item *p_i_* appears in the higher-ranked rules. The value of *f*(*d, p_i_, r*) is always between 0 and 1. Consequently, the value of mean(*f*(*r*)) is always between 0 and 1.
5. The term *δ_actionable_*(*r*) is the indicator function for whether *r* is actionable, has a weight *w_a_*>0, and addresses factor 5.

Let *v_r_*(*x*) denote the variable, such as confidence, whose value on the association rule *r* is *x*. min(*v_r_*(*x*)) and max(*v_r_*(*x*)) denote the minimum and maximum values of *v_r_*(*x*) across all the rules found for the patient, respectively. If max(*v_r_*(*x*))≠min(*v_r_*(*x*)), the function norm(*x*) 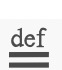 [*x−*min(*v_r_*(*x*))]/[max(*v_r_*(*x*))*−*min(*v_r_*(*x*))] normalizes *x* to a value between 0 and 1. If max(*v_r_*(*x*))=min(*v_r_*(*x*)), all of the rules found for the patient have the same value of *v_r_*(*x*), and thus, there is no need to consider *v_r_*(*x*) in ranking these rules. In this case, norm(*x*) is set to 0.

*C_r_*, log_10_*S_r_*, and *N_r_* have different value ranges. To make *C_r_*, log_10_*S_r_*, and *N_r_* comparable with each other, we use norm() to put them into the same range of 0 to 1. mean(*f*(*r*)) and *δ_actionable_*(*r*) also fall within this range. To reflect that factors 1, 2, and 3 are equally important, we set the default values of *w_c_*, *w_s_*, and *w_n_* to 1. To encourage the top-ranked rules to include diversified feature-value pair items, we wanted *w_d_*’s value to be >1 and set *w_d_*’s default value to 50. To strongly push the actionable rules to rank higher than the nonactionable rules, we wanted *w_a_*’s value to be ≫1 and set *w_a_*’s default value to 100. The value of *w_a_* does not impact the score differences and, hence, the relative rankings among the actionable rules. When *w_a_* is >*w_c_*+*w_s_*+*w_n_*+*w_d_*, the actionable rules always have larger scores than the nonactionable rules because norm(*C_r_*), norm(log_10_*S_r_*), norm(*N_r_*), and mean(*f*(*r*)) are all between 0 and 1.

#### Detailed Description of the Five Terms Used in the Rule Scoring Function

In this section, we sequentially describe the five terms used in the rule scoring function in detail.

As norm() is a monotonically increasing function, all else being equal, the term norm(*C_r_*) gives a larger ranking score to an association rule with a higher confidence *C_r_*.

**Figure 2:**
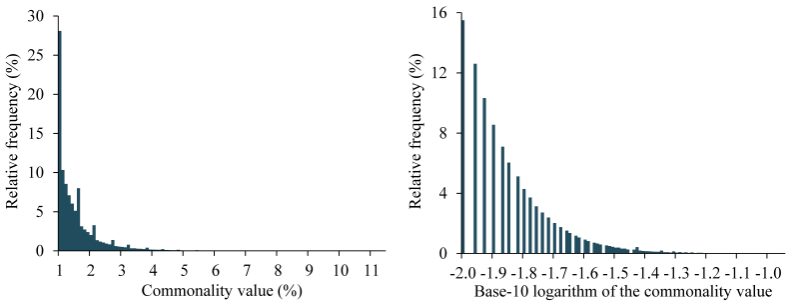
The distribution of the commonality values of all of the association rules used by our automated explanation method for predicting asthma hospital encounters in patients with asthma at the University of Washington Medicine.

As shown in Figure 2, the commonality values for the association rules used by our automated explanation method have a skewed distribution. Most of the commonality values are clustered in the lower-value range. The commonality values of the rules generated by our automated explanation method for a patient are a sample from this distribution. We want the same weight *w_s_* to work for different patients, regardless of how the sample is taken from this distribution. Thus, for every patient, we want the variance of the terms computed on the corresponding rules’ commonality values to have approximately the same scale. For this purpose, we use the log_10_() function to transform the commonality values so that the resulting values are distributed more evenly than the raw values. As both norm() and log_10_() are monotonically increasing functions, norm(log_10_()) is also a monotonically increasing function. All else being equal, the term norm(log_10_*S_r_*) gives a larger ranking score to a rule with a higher commonality *S_r_*.

As −norm() is a monotonically decreasing function, all else being equal, the term −norm(*N_r_*) assigns a larger ranking score to an association rule with a smaller number *N_r_* of feature-value pair items on its left-hand side.

In the *k*-th iteration of the association rule ranking process, the top *k*−1 rules have already been determined. We work on identifying the *k*-th ranked rule. For each feature-value pair item *p_i_* on the left-hand side of a rule *r* that is found for the patient and whose rank has not yet been decided, we compute the exponential decay function *f*(*d*, *p_i_*, *r*) 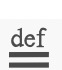 exp(−*d*·*t_i_*). Here, *d*>0 is the decay constant, with a default value of 5. *t_i_* is the number of times *p_i_* appears in the top *k*−1 rules. A larger value of *t_i_* results in a smaller value of *f*(*d*, *p_i_*, *r*). Recall that the term mean(*f*(*r*)) is the mean of *f*(*d*, *p_i_*, *r*) over all the items on *r*’s left-hand side. All else being equal, mean(*f*(*r*)) assigns a smaller ranking score to a rule whose left-hand side has more items appearing in the top *k*−1 rules.

*δ_actionable_*(*r*) is equal to 1 if the association rule *r* is actionable and is equal to 0 if *r* is nonactionable. All else being equal, the term *δ_actionable_*(*r*) assigns a larger ranking score to an actionable rule compared with that of a nonactionable rule.

#### The Iterative Association Rule Ranking Process

If only one association rule is found for a patient, there is no need to rank the rule. If ≥2 rules are found for the patient, we rank these rules iteratively. In the *k*-th iteration, we compute the ranking score for every rule *r* that is found for the patient and whose rank has not yet been determined. Compared with the case in the previous iteration, the score needs to be updated if and only if the value of mean(*f*(*r*)) changes, that is, if and only if any feature-value pair item on *r*’s left-hand side also appears on the left-hand side of the (*k*−1)-th ranked rule. Among all the rules that are found for the patient and whose ranks have not yet been determined, we select the rule with the highest score as the *k*-th ranked rule. If ≥2 of these rules have the same highest score, we choose one of them randomly as the *k*-th ranked rule.

#### For Each Association Rule on Display, Sort the Feature-Value Pair Items on Its Left-Hand Side

The same feature-value pair item could appear on the left-hand side of ≥2 top-ranked association rules. The user of the automated explanation function tends to read both the rules and the items on the left-hand side of a rule in the display order. To help the user obtain the most useful information as quickly as possible, for each rule on display, we need to appropriately rank the items on its left-hand side. For this purpose, we considered two factors:

1. *Factor 6*: The user wants to see new information as quickly as possible. Hence, all else being equal, an item for a rule that already appears in the higher-ranked rules should rank lower. As the number of times the item appears in higher-ranked rules increases, the rank of the item should decrease.
2. *Factor 7*: The user wants to find suitable interventions for the patient. Thus, all else being equal, an actionable item should rank higher than a nonactionable item.

We incorporate the two factors listed above into an item scoring function to strike a balance between them. Consider the *k*-th ranked association rule. For each feature-value pair item *p* on its left-hand side, *p*’s ranking score is a linear combination of two terms, one per factor:

*score_p_=w_g_·*exp(*−d·t*)*+w_b_·*δ*_actionable_*(*p*) ** (5)**

The terms in the equation above are further explained below:

1. In the equation for *score_p_* above, *d* is the same decay constant used in *f*(*d, p_i_, r*) in the rule scoring function. *t* is the number of times *p* appears in the top *k*−1 rules. The larger the value of *t*, the smaller the value of the exponential decay function exp(−*d·t*). Hence, all else being equal, the exp(−*d·t*) term assigns a smaller ranking score to an item that appears more times in the top *k*−1 rules. This addresses factor 6.
2. The term *δ_actionable_*(*p*) is an indicator function for whether *p* is actionable. The term *δ_actionable_*(*p*) is equal to 1 if *p* is actionable and is equal to 0 if *p* is nonactionable. All else being equal, the *δ_actionable_*(*p*) term causes an actionable item to have a higher ranking score than that of a nonactionable item. This addresses factor 7.

Both exp(−*d*·*t*) and *δ_actionable_*(*p*) are between 0 and 1. For the weight *w_g_*>0 of the term exp(−*d*·*t*), we set its default value to 1. For the weight *w_b_*>0 of the term *δ_actionable_*(*p*), we set its default value to 2, which is >1. The value of *w_b_* has no impact on the score differences and, hence, the relative ranking among the actionable items on the left-hand side of the association rule. When *w_b_* is >*w_g_*, the actionable items always have larger scores than those of the nonactionable items because exp(−*d*·*t*) is between 0 and 1.

When the rank of an association rule is decided, we compute the ranking score for each feature-value pair item on the rule’s left-hand side. We then sort these items in descending order of their scores. Items with the same score are randomly prescribed and given consecutive ranks.

### Computer Coding Implementation

We used the R programming language to implement our explanation ranking method.

### Providing Informative Examples of the Explanation Ranking Results

We want to demonstrate various aspects of the results produced by our explanation ranking method. For this purpose, we chose 8 patients with asthma in the test set, each of whom our UWM model correctly predicted to have ≥1 asthma hospital encounter in 2019, and our automated explanation method could explain this prediction. For each patient, we show the top three explanations produced by our explanation ranking method. Each patient satisfied one or more of the following conditions and was an informative case:

1. *Condition 1*: The patient had numerous encounters, laboratory tests, or medication prescriptions in 2018, reflecting a complex condition. In this case, we want to show how well the top three explanations capture and summarize the patient’s key information related to asthma outcome prediction.
2. *Condition 2*: All or most of the asthma-related encounters that the patient had in 2018 were ED visits. Such a patient often had poor asthma control because of poor treatment adherence. In this case, we want to show how well the interventions linking to the top three explanations address the poor asthma control.
3. *Condition 3*: For each of the top three association rules produced for the patient, the rule’s confidence value is close to the minimum confidence threshold. The rule’s commonality value is close to the minimum commonality threshold. In this case, we want to illustrate these *borderline* rules. Recall that below either threshold, a rule will not be used by our automated explanation method.
4. *Condition 4*: The top three rules produced for the patient share several common feature-value pair items on their left-hand sides. This could happen, for example, when our automated explanation method finds only a few rules for the patient because the patient had only a small amount of information recorded in the electronic health record (EHR) system during the past year. In this case, we want to demonstrate the information redundancy in these rules.
5. *Condition 5*: A patient at high risk for future asthma hospital encounters often had ≥1 hospital encounter related to asthma during the past year. The patient being examined does not fall into this category. The patient had several feature values correlated with future asthma hospital encounters but no hospital encounter related to asthma during the past year. In this case, we want to show how well the top three explanations capture these feature values.

### Sensitivity Analysis of the Parameters Used in the Rule Scoring Function

The rule scoring function uses six parameters whose default values are as follows: *w_c_*=1, *w_s_*=1, *w_n_*=1, *w_d_*=50, *d=*5, and *w_a_*=100. To assess the impact of the five parameters *w_c_*, *w_s_*, *w_n_*, *w_d_*, and *d* on the association rule ranking results, we performed five experiments. In each experiment, we changed the value of one of these five parameters and kept the other parameters at their default values. In comparison with the case of all parameters taking their default values, we measured the average percentage change in the unique feature-value pair items contained in the top min(3, *q*) rules for a patient, where *q* denotes the number of rules generated by our automated explanation method for the patient. The percentage change in the unique items was defined as 100×the number of changed unique items divided by the number of unique items in the top min(3, *q*) rules. The average was taken over all patients in the test set, each of whom was predicted to have ≥1 asthma hospital encounter in 2019 and had at least one applicable rule (ie, *q≥*1). Multiple rules often differ from each other by only one item on their left-hand sides. In addition, switching items among the top few rules for a patient has little impact on the total amount of information that the user of the automated explanation function obtains from these rules. Thus, we measured the number of changed unique items in the top few rules per patient instead of the number of changed top rules per patient or the number of changed items per top rule.

As explained before, when *w_a_* is >*w_c_*+*w_s_*+*w_n_*+*w_d_*, the actionable rules always rank higher than the nonactionable rules. Meanwhile, the concrete value of *w_a_* has no impact on the ranking of the actionable rules. All the rules that our automated explanation method used on the UWM data set were actionable [27]. Thus, we did not perform a sensitivity analysis on *w_a_*. For a similar reason, we did not perform a sensitivity analysis on the weights *w_g_* and *w_b_* used in the item scoring function.

## Results

### The Demographic and Clinical Characteristics of Our Patient Cohort

Each UWM data instance used in this study corresponds to a distinct patient and index year pair and is used to predict the patient’s outcome in the succeeding 12 months. Tables S1 and S2 in Multimedia Appendix 1 show our patient cohort’s demographic and clinical characteristics during 2011-2017 and 2018 separately. These two sets of characteristics were similar to each other. During 2011-2017, 1.74% (1184/68,244) of data instances were linked to asthma hospital encounters in the succeeding 12 months. During 2018, 1.49% (218/14,644) of data instances were linked to asthma hospital encounters in the succeeding 12 months. A detailed comparison of these two sets of characteristics is presented in our previous paper [12].

### Execution Time

For an average patient with asthma, our explanation ranking method took <0.01 seconds to produce the top three explanations. This is sufficiently fast for providing real-time clinical decision support.

### Informative Examples of the Explanation Ranking Results

#### The Top Three Association Rules That Our Explanation Ranking Method Produced in Each Informative Example

The test set included 134 patients with asthma, each of whom our UWM model correctly predicted to have ≥1 asthma hospital encounter in 2019, and our automated explanation method could explain this prediction. To show the reader various aspects of the results produced by our explanation ranking method, we chose 8 of these patients who were informative cases. Tables 1-8 present the top three association rules that our explanation ranking method produced for each of the eight patients. For each of the top three rules produced for the seventh selected patient, Table 9 lists the interventions linked to the rule.

**Table 1:** The top three association rules that our explanation ranking method produced for the first selected patient (patient 1). This patient satisfied condition 1.

Rank	Association rule	Confidence of the rule			Commonality of the rule (n=1184), n (%)
		Total, n	Value, n (%)		
1	The patient had 2 or 3 ED^a^ visits related to asthma during the past year AND the patient was prescribed between 7 and 11 distinct asthma medications during the past year AND the patient was prescribed between 5 and 7 distinct asthma relievers during the past year AND the patient had ≥1 active problem in the problem list during the past year → The patient will likely have ≥1 inpatient stay or ED visit for asthma in the succeeding 12 months.	46	24 (52.17)	24 (2.03)	
2	The patient’s mean length of stay of an ED visit during the past year was >0.205 day AND the patient was prescribed ≥4 systemic corticosteroids during the past year AND the patient’s most recent ED visit related to asthma occurred no less than 26 days ago and no more than 100 days ago AND the patient was prescribed 2 distinct nebulizer medications during the past year AND the patient is not a White patient → The patient will likely have ≥1 inpatient stay or ED visit for asthma in the succeeding 12 months.	28	14 (50)	14 (1.18)	
3	The patient was prescribed nebulizer medications ≥8 times during the past year AND the patient had ≥5 no shows during the past year AND the patient had 2 or 3 ED visits related to asthma during the past year AND the patient’s mean temperature during the past year was ≤98.09 Fahrenheit AND the patient is ≤54 years old → The patient will likely have ≥1 inpatient stay or ED visit for asthma in the succeeding 12 months.	32	18 (56.25)	18 (1.52)	

^a^ED: emergency department.

**Table 2:** The top three association rules that our explanation ranking method produced for the second selected patient (patient 2). This patient satisfied condition 1.

Rank	Association rule	Confidence of the rule			Commonality of the rule (n=1184), n (%)
		Total, n	Value, n (%)		
1	The patient’s most recent diagnosis of asthma with acute exacerbation or status asthmaticus was from ≤110 days ago AND the patient was prescribed ≥10 short-acting *β*-2 agonists during the past year AND the patient had no outpatient visit during the past year AND the patient’s first encounter related to asthma was from ≥1 year ago → The patient will likely have ≥1 inpatient stay or ED^a^ visit for asthma in the succeeding 12 months.	87	54 (62.07)	54 (4.56)	
2	The patient was prescribed asthma medications ≥16 times during the past year AND the patient’s mean respiratory rate during the past year was >16.89 breaths per minute AND the patient’s most recent visit was an ED visit AND the patient is a Black or an African American patient AND the patient was totally allowed between 1 and 33 medication refills during the past year → The patient will likely have ≥1 inpatient stay or ED visit for asthma in the succeeding 12 months.	32	18 (56.25)	18 (1.52)	
3	The patient had between 8 and 16 asthma diagnoses during the past year AND the patient’s lowest SpO_2_^b^ level during the past year was between 8.0% and 94.5% AND the patient’s most recent ED visit related to asthma occurred no less than 26 days ago and no more than 100 days ago AND the patient is not a White patient AND the patient had ≤6 encounters during the past year → The patient will likely have ≥1 inpatient stay or ED visit for asthma in the succeeding 12 months.	35	18 (51.43)	18 (1.52)	

^a^ED: emergency department.

^b^SpO_2_: peripheral capillary oxygen saturation.

**Table 3:** The top three association rules that our explanation ranking method produced for the third selected patient (patient 3). This patient satisfied condition 1.

Rank	Association rule	Confidence of the rule			Commonality of the rule (n=1184), n (%)
		Total, n	Value, n (%)		
1	The patient’s most recent diagnosis of asthma with acute exacerbation or status asthmaticus was from ≤110 days ago AND the patient’s most recent visit was an ED^a^ visit AND the patient had between 9 and 17 primary or principal asthma diagnoses during the past year → The patient will likely have ≥1 inpatient stay or ED visit for asthma in the succeeding 12 months.	127	79 (62.2)	79 (6.67)	
2	The patient had between 17 and 27 asthma diagnoses during the past year AND the patient’s most recent visit was an ED visit AND the patient had no visit to the primary care provider during the past year → The patient will likely have ≥1 inpatient stay or ED visit for asthma in the succeeding 12 months.	68	38 (55.88)	38 (3.21)	
3	The patient was prescribed ≥10 short-acting *β*-2 agonists during the past year AND the highest severity of all asthma diagnoses of the patient during the past year was moderate or severe persistent asthma AND the patient was allowed ≥34 medication refills during the past year AND the patient is ≤54 years old → The patient will likely have ≥1 inpatient stay or ED visit for asthma in the succeeding 12 months.	40	20 (50)	20 (1.69)	

^a^ED: emergency department.

**Table 4:** The top three association rules that our explanation ranking method produced for the fourth selected patient (patient 4). This patient satisfied condition 2.

Rank	Association rule	Confidence of the rule			Commonality of the rule (n=1184), n (%)
		Total, n	Value, n (%)		
1	The patient had ≥7 ED^a^ visits related to asthma during the past year AND the patient is single → The patient will likely have ≥1 inpatient stay or ED visit for asthma in the succeeding 12 months.	37	34 (91.89)	34 (2.87)	
2	The patient had between 9 and 17 primary or principal asthma diagnoses during the past year AND the patient’s most recent outpatient visit related to asthma was from ≥365 days ago → The patient will likely have ≥1 inpatient stay or ED visit for asthma in the succeeding 12 months.	105	66 (62.86)	66 (5.57)	
3	The patient had ≥28 asthma diagnoses during the past year AND the patient had no outpatient visit during the past year → The patient will likely have ≥1 inpatient stay or ED visit for asthma in the succeeding 12 months.	19	16 (84.21)	16 (1.35)	

^a^ED: emergency department.

**Table 5:** The top three association rules that our explanation ranking method produced for the fifth selected patient (patient 5). This patient satisfied condition 5.

Rank	Association rule	Confidence of the rule			Commonality of the rule (n=1184), n (%)
		Total, n	Value, n (%)		
1	The patient had ≥20 diagnoses of asthma with acute exacerbation during the past year AND the patient was prescribed ≥10 short-acting *β*-2 agonists during the past year → The patient will likely have ≥1 inpatient stay or ED^a^ visit for asthma in the succeeding 12 months.	82	48 (58.54)	48 (4.05)	
2	The patient had ≥28 asthma diagnoses during the past year AND the patient was prescribed nebulizer medications ≥8 times during the past year AND the patient had no outpatient visit to the primary care provider during the past year → The patient will likely have ≥1 inpatient stay or ED visit for asthma in the succeeding 12 months.	55	37 (67.27)	37 (3.13)	
3	The patient had ≥18 primary or principal asthma diagnoses during the past year AND the patient was prescribed ≥8 distinct asthma relievers during the past year AND the patient’s mean heart rate during the past year was >80 beats per minute → The patient will likely have ≥1 inpatient stay or ED visit for asthma in the succeeding 12 months.	116	58 (50)	58 (4.9)	

^a^ED: emergency department.

**Table 6:** The top three association rules that our explanation ranking method produced for the sixth selected patient (patient 6). This patient satisfied conditions 3 and 4.

Rank	Association rule	Confidence of the rule			Commonality of the rule (n=1184), n (%)
		Total, n	Value, n (%)		
1	The patient had 2 or 3 ED^a^ visits related to asthma during the past year AND the patient’s most recent outpatient visit related to asthma was from ≤104 days ago AND the patient was prescribed ≤2 inhaled corticosteroids during the past year AND the patient is ≤54 years old AND the patient’s relative change of weight during the past year was ≤3% → The patient will likely have ≥1 inpatient stay or ED visit for asthma in the succeeding 12 months.	40	22 (55)	22 (1.86)	
2	The patient had between 3 and 8 diagnoses of asthma with (acute) exacerbation during the past year AND the patient had 2 or 3 ED visits related to asthma during the past year AND the patient is not a White patient AND the patient was prescribed ≤2 distinct asthma medications during the past year AND the patient is single → The patient will likely have ≥1 inpatient stay or ED visit for asthma in the succeeding 12 months.	25	14 (56)	14 (1.18)	
3	The patient’s most recent outpatient visit related to asthma was from ≤104 days ago AND the patient had 2 or 3 ED visits related to asthma during the past year AND the patient was prescribed ≥1 unit of medications during the past year AND the patient had no public insurance on the last day of the past year AND the patient had between 1 and 13 outpatient visits during the past year → The patient will likely have ≥1 inpatient stay or ED visit for asthma in the succeeding 12 months.	32	16 (50)	16 (1.35)	

^a^ED: emergency department.

**Table 7:** The top three association rules that our explanation ranking method produced for the seventh selected patient (patient 7). This patient satisfied conditions 1 and 2.

Rank	Association rule	Confidence of the rule			Commonality of the rule (n=1184), n (%)
		Total, n	Value, n (%)		
1	The patient had ≥7 ED^a^ visits related to asthma during the past year → The patient will likely have ≥1 inpatient stay or ED visit for asthma in the succeeding 12 months.	51	39 (76.47)	39 (3.29)	
2	The patient had between 17 and 27 asthma diagnoses during the past year AND the patient had no outpatient visit during the past year → The patient will likely have ≥1 inpatient stay or ED visit for asthma in the succeeding 12 months.	48	28 (58.33)	28 (2.36)	
3	The patient’s mean length of stay of an ED visit during the past year was between 0.025 and 0.205 day AND the patient had ≥3 ED visits during the past year AND the patient was prescribed ≥3 asthma relievers that are neither short-acting *β*-2 agonists nor systemic corticosteroids during the past year AND the patient was prescribed ≥4 systemic corticosteroids during the past year AND the patient is single → The patient will likely have ≥1 inpatient stay or ED visit for asthma in the succeeding 12 months.	116	58 (50)	58 (4.9)	

^a^ED: emergency department.

**Table 8:** The top three association rules that our explanation ranking method produced for the eighth selected patient (patient 8). This patient satisfied condition 5.

Rank	Association rule	Confidence of the rule			Commonality of the rule (n=1184), n (%)
		Total, n	Value, n (%)		
1	The patient had between 9 and 17 primary or principal asthma diagnoses during the past year AND the patient was prescribed asthma medications ≥16 times during the past year AND the patient had no outpatient visit to the primary care provider during the past year AND the patient is not a White patient → The patient will likely have ≥1 inpatient stay or ED^a^ visit for asthma in the succeeding 12 months.	87	45 (51.72)	45 (3.8)	
2	For the patient’s most recent visit, the time from making the request to the actual visit was ≤0.6 day AND the patient was prescribed asthma medications ≥16 times during the past year AND the patient is a Black or an African American patient AND the patient’s first encounter related to asthma was from ≥1 year ago AND the patient’s lowest SpO_2_^b^ level during the past year was between 94.5% and 95.5% → The patient will likely have ≥1 inpatient stay or ED visit for asthma in the succeeding 12 months.	19	12 (63.16)	12 (1.01)	
3	The patient was prescribed ≥12 distinct asthma medications during the past year AND the patient had ≥12 encounters during the past year AND the patient’s most recent outpatient visit related to asthma was from ≤104 days ago AND the patient had ≤82 laboratory tests during the past year AND the patient is not a White patient → The patient will likely have ≥1 inpatient stay or ED visit for asthma in the succeeding 12 months.	19	12 (63.16)	12 (1.01)	

^a^ED: emergency department.

^b^SpO_2_: peripheral capillary oxygen saturation.

**Table 9:** The interventions linked to each of the top three association rules that our explanation ranking method produced for patient 7.

Rank	Association rule	Linked interventions
1	The patient had ≥7 ED^a^ visits related to asthma during the past year → The patient will likely have ≥1 inpatient stay or ED visit for asthma in the succeeding 12 months.	An intervention linked to the item “the patient had ≥7 ED visits related to asthma during the past year” is to use control strategies to prevent needing emergency care.
2	The patient had between 17 and 27 asthma diagnoses during the past year AND the patient had no outpatient visit during the past year → The patient will likely have ≥1 inpatient stay or ED visit for asthma in the succeeding 12 months.	An intervention linked to the item “the patient had between 17 and 27 asthma diagnoses during the past year” is to give the patient suggestions on how to improve asthma control. An intervention linked to the item “the patient had no outpatient visit during the past year” is to make sure that the patient has a primary care provider and to suggest the patient to regularly visit the provider.
3	The patient’s mean length of stay of an ED visit during the past year was between 0.025 and 0.205 day AND the patient had ≥3 ED visits during the past year AND the patient was prescribed ≥3 asthma relievers that are neither short-acting *β*-2 agonists nor systemic corticosteroids during the past year AND the patient was prescribed ≥4 systemic corticosteroids during the past year AND the patient is single → The patient will likely have ≥1 inpatient stay or ED visit for asthma in the succeeding 12 months.	An intervention linked to the items “the patient’s mean length of stay of an ED visit during the past year was between 0.025 and 0.205 day” and “the patient had ≥3 ED visits during the past year” is to use control strategies to prevent needing emergency care. An intervention linked to the items “the patient was prescribed ≥3 asthma relievers that are neither short-acting *β*-2 agonists nor systemic corticosteroids during the past year” and “the patient was prescribed ≥4 systemic corticosteroids during the past year” is to tailor the prescribed asthma medications, to help the patient adhere to asthma controllers, and to improve avoidance of triggers.

^a^ED: emergency department.

As illustrated by the cases shown in Tables 1-9, the top few explanations that our explanation ranking method produces for a patient offer five benefits for clinical decision support. We describe these five benefits sequentially in the following sections.

#### Benefit 1: The Top Few Explanations Provide Succinct Summaries on a Wide Range of Aspects of the Patient’s Situation

To make good clinical decisions for a patient, the clinician needs to understand the patient’s situation well. For each of the eight selected patients, the top three rule-based explanations produced by our explanation ranking method provide succinct summaries on a wide range of aspects of the patient’s situation, such as demographics, encounters, vital signs, laboratory tests, and medications. From these summaries, the user of the automated explanation function can quickly gain a comprehensive understanding of the patient’s situation related to the prediction target. This saves the user a significant amount of time and effort. In comparison, to gain this understanding in a clinical setting, even if a clinician knows all of the features needed for this purpose, the clinician currently often needs to spend a significant amount of time laboriously checking many pages of information scattered in various places in the EHR system and performing manual calculations. For example, patient 1 had a total of >1000 encounters recorded in the EHR system at the UWM over time. In 2018, this patient had 164 encounters, only two of which were related to asthma, and both were ED visits. As Table 1 shows, the statistics of two ED visits related to asthma are reflected by the first item on the left-hand side of the first association rule produced for this patient. As another example, in 2018, patient 2 had 740 medication prescriptions, 153 of which were asthma medication prescriptions covering a total of 72 short-acting *β*-2 agonists. As Table 2 shows, the statistic of 72 short-acting *β*-2 agonists is reflected by the first item on the left-hand side of the first rule produced for this patient. The statistics of 153 asthma medication prescriptions are reflected by the first item on the left-hand side of the second rule produced for this patient. The cases with the other items on the left-hand sides of the top three rules produced for these two patients were similar.

To gain a comprehensive understanding of a patient’s situation quickly, a clinician could ask the patient to describe his or her situation. However, the patient often cannot perform this well. For example, patients 1, 3, and 7 had severe mental disorders, which affected their memory and ability to describe their situation. This was a common scenario. Over 29.99% (4393/14,644) of patients with asthma at the UWM have mental disorders. Moreover, when making clinical decisions, the clinician does not always have direct access to the patient. For instance, when identifying candidate patients for care management, care managers are sitting in a back office and cannot talk to patients. In either of these two cases, the summaries provided by the top few rule-based explanations can help the clinician gain an understanding of the patient.

#### Benefit 2: Showing the Top Few Explanations Can Save the User of the Automated Explanation Function From Having to Manually Think of Many Features Summarizing the Patient’s Situation and Computing Their Values

Often, many features must be used to adequately summarize a patient’s situation related to the prediction target. In a busy clinical environment, a clinician cannot be expected to enumerate all of these features in a short amount of time. The top few rule-based explanations that our explanation ranking method produces for a patient cover the values of various features summarizing the patient’s situation related to the prediction target. This saves the user of the automated explanation function from having to manually think of these features and to compute their values.

#### Benefit 3: The Top Few Explanations Can Provide Information Not Easily Obtainable From Using the Existing Search and Browsing Functions of the EHR System to Check the Patient’s Data

The EHR system provides some browsing and basic search functions. However, for certain important features summarizing a patient’s situation related to the prediction target, we cannot easily obtain their values by using these functions to check the patient’s EHR data. The top few rule-based explanations that our explanation ranking method produces for a patient cover the values of several such features. This saves the user of the automated explanation function a significant amount of work. For example, many different asthma medications exist. In 2018, patient 2 had 740 medication prescriptions. It is difficult and time-consuming to manually compute the number of asthma medication prescriptions and the total number of short-acting *β*-2 agonists prescribed for this patient in 2018. In comparison, as mentioned before, these two statistics are directly reflected by the first and second rules produced for this patient. As a second example, in 2018, patient 7 had 14 ED visits, eight of which were related to asthma. For two of these eight ED visits, asthma was not the primary diagnosis. To compute the patient’s number of ED visits related to asthma in 2018, a clinician needs to find all of the patient’s ED visits in 2018 and check each of them to see whether it has an asthma diagnosis code. This requires a nontrivial amount of time. In comparison, as Table 7 shows, the statistics of eight ED visits related to asthma are directly reflected by the first item on the left-hand side of the first rule produced for this patient. As a third example, in 2018, patient 8 had 12 outpatient visits, none of which was to the patient’s primary care provider. To compute the patient’s number of outpatient visits to the primary care provider, a clinician needs to find all of the patient’s outpatient visits in 2018 and manually check each of them to see whether it involved the patient’s primary care provider. This requires a nontrivial amount of time. In comparison, as Table 8 shows, the third item on the left-hand side of the first rule produced for this patient directly shows that the patient had 0 outpatient visits to the primary care provider in 2018.

#### Benefit 4: The Top Few Explanations Can Help the User of the Automated Explanation Function Avoid Overlooking Certain Important Information of the Patient and Discover Errors in the Data Recorded on the Patient in the EHR System

A patient with asthma often has several other diseases, which could distract the clinicians and cause them to pay insufficient attention to the patient’s asthma and record incorrect data on the patient in the EHR system. For example, in 2018, asthmatic patient 3 also had major depression disorder, anxiety, posttraumatic stress disorder, visual disturbance, chronic pain, and knee osteoarthritis. In the patient’s problem list, these diseases were recorded as major problems, whereas asthma was recorded as a minor problem. However, the patient had 15 primary asthma diagnoses, some of which were severe persistent asthma and indicated that asthma was a major problem for the patient at that time. In 2020, asthma was first recorded as two major problems in the patient’s problem list: one on asthma exacerbation and another on persistent asthma with status asthmaticus. As shown in Table 3, the first and third rules produced for the patient covered the patient’s number of asthma diagnoses and the highest severity of these diagnoses in 2018, reflecting that the patient had severe persistent asthma at that time. This can help the user of the automated explanation function avoid overlooking this aspect and discover that asthma should be recorded as a major problem in the patient’s problem list in 2018.

#### Benefit 5: The Top Few Explanations Can Help the User of the Automated Explanation Function Identify Certain Problems of the Patient Not Easily Findable in the EHR System

This can help the user of the automated explanation function identify suitable interventions for the patient. For example, as shown in Table 6, the first and second rules produced for patient 6 showed that this patient had quite a few ED visits related to asthma; however, very few asthma medications were prescribed for this patient in 2018. This patient did not adhere to albuterol prescriptions due to personal preference. Realizing this, the user could consider adopting the intervention of replacing albuterol with some other asthma medications that the patient is willing to take. As another example, as shown in Tables 4 and 7, for patients 4 and 7, the top three rules produced for each patient revealed that the patient had many ED visits related to asthma but no outpatient visit in 2018. These two patients were found to be homeless. With this information, the user could consider providing social resources to reduce the socioeconomic burden of homelessness, which leads to ineffective access to health care.

#### Description of the 5 Example Patient Cases, One Case Per Each of Conditions 1-5

In this section, for each of conditions 1-5, we choose one example patient satisfying it and show how this patient was an informative case.

As an example case for condition 1, patient 1 had 164 encounters and 644 medication prescriptions in 2018. As shown in Table 1, the top three explanations produced for this patient effectively capture and summarize various aspects of the patient’s key information related to future asthma hospital encounters.

As an example case for condition 2, patient 7 had eight asthma-related encounters in 2018, all of which were ED visits. As shown in Table 7, the top three explanations produced for this patient revealed that the patient had many asthma diagnoses, had no outpatient visit, and was prescribed ≥4 systemic corticosteroids during 2018, reflecting poor asthma control. As shown in Table 9, the interventions linked to the top three explanations address various aspects related to poor asthma control.

Patient 6 provides an example for condition 3. As shown in Table 6, for each of the top three association rules produced for this patient, the rule’s confidence value is close to the minimum confidence threshold of 50%, and the rule’s commonality value is close to the minimum commonality threshold of 1%. These three rules cover a wide range of aspects of the patient’s situation, including demographics, encounters, diagnoses, vital signs, and medications.

As an example case for condition 4, patient 6 had only three encounters and one medication order, and subsequently, a small amount of information was recorded for this patient in the EHR system in 2018. As shown in Table 6, the top three explanations produced for this patient share three common feature-value pair items on their left-hand sides. Despite having moderate information redundancy, these explanations still cover a wide range of aspects of the patient’s situation, including demographics, encounters, diagnoses, vital signs, and medications.

As an example case for condition 5, patient 8 had no hospital encounters related to asthma in 2018. As shown in Table 8, the top three explanations produced for this patient capture several feature values of the patient correlated with future asthma hospital encounters, such as the patient having between 9 and 17 primary or principal asthma diagnoses during the past year, the patient having ≥16 asthma medication prescriptions during the past year, the patient having no outpatient visit to the primary care provider during the past year, and the patient having ≥12 encounters during the past year.

### Sensitivity Analysis Results of the Parameters Used in the Rule Scoring Function

**Figure 3:**
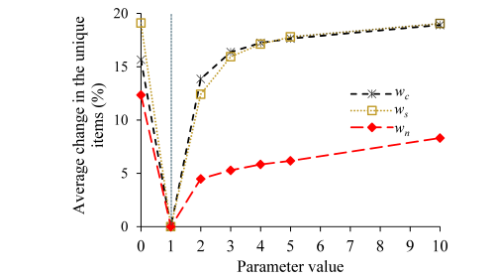
In comparison with the case where all five parameters took their default values and for each of the three parameters *w_c_*, *w_s_*, and *w_n_*, the average percentage change in the unique feature-value pair items contained in the top min (3, *q*) association rules for a patient versus the parameter’s value. The vertical dotted line represents the default value of *w_c_*, *w_s_*, and *w_n_*.

**Figure 4:**
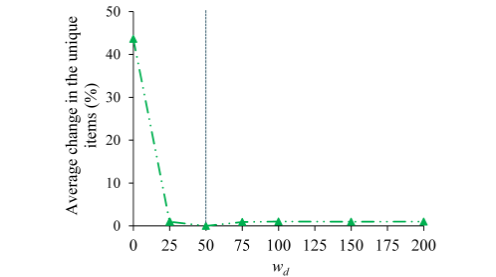
In comparison with the case where all five parameters took their default values, the average percentage change in the unique feature-value pair items contained in the top min (3, *q*) association rules for a patient versus the value of the parameter *w_d_*. The vertical dotted line represents the default value of *w_d_*.

**Figure 5:**
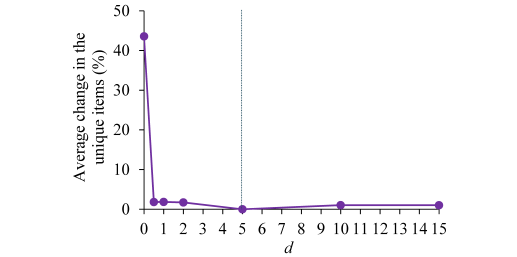
In comparison with the case where all five parameters took their default values, the average percentage change in the unique feature-value pair items contained in the top min (3, *q*) association rules for a patient versus the value of the parameter *d*. The vertical dotted line represents the default value of *d*.

We performed 5 sensitivity analysis experiments, 1 for each of the 5 parameters *w_c_*, *w_s_*, *w_n_*, *w_d_*, and *d* used in the rule scoring function. In each experiment, we changed the corresponding parameter’s value and kept the other parameters at their default values. In comparison with the case where all 5 parameters took their default values and for each of these 5 parameters, Figures 3-5 show the average percentage change in the unique feature-value pair items contained in the top min(3, *q*) association rules for a patient versus the parameter’s value. In each figure, the vertical dotted line represents the default value of the corresponding parameter. For each parameter value tested, the average percentage change in the unique items was relatively small (<20%). The only exception is the case of either *w_d_*=0 or *d*=0, where the average percentage change in the unique items was 43.57% (453.18/1040). In both cases, our explanation ranking method ignores the need for the top-ranked rules to provide diversified information (factor 4).

## Discussion

### Principal Findings

In a busy clinical environment, the explanation ranking module is essential for our automated explanation function for machine learning predictions to provide high-quality real-time decision support. For an average patient with asthma correctly predicted by our UWM model to have future asthma hospital encounters, our automated explanation method generated over 5000 rule-based explanations, if any. Within a negligible amount of time, our explanation ranking method can appropriately rank them and return the few highest-ranked explanations. These few explanations typically have high quality and low redundancy. From these few explanations, the user of the automated explanation function can gain useful insights on various aspects of the patient’s situation. Many of these insights cannot be easily obtained by viewing the patient’s data in the current EHR system. With further improvements in model accuracy, our UWM model coupled with our automated explanation method and our explanation ranking method could be deployed to better guide the use of asthma care management to save costs and improve patient outcomes.

Similar to our automated explanation method, our explanation ranking method is general purpose and does not rely on any specific property of a particular prediction target, disease, patient cohort, or health care system. Our automated explanation method coupled with our explanation ranking method can be used for any predictive modeling problem on any tabular data set. This provides a unique solution to the interpretability issue that deters the widespread adoption of machine learning predictive models in clinical practice.

In our sensitivity analysis, when we changed any parameter used in our explanation ranking method from its default value, the resulting average percentage change in the unique feature-value pair items contained in the top min(3, *q*) association rules for a patient was typically <20%. This is not a large change, as most (>80%) of the distinct feature-value pair items contained in these rules and, subsequently, most of the information seen by the user of the automated explanation function remain the same. For instance, if the top min(3, *q*) association rules contain 15 unique feature-value pair items, at most three of these feature-value pair items would vary due to the change in the parameter value, whereas the other 12 or more remain the same as before. Thus, each parameter used in our explanation ranking method has a reasonably large stable range, within which the top few explanations produced by our method do not vary greatly as the parameter value changes. The default value of the parameter was within this stable range. According to our test results, the stable ranges are 0 to 10 for *w_c_*, 0 to 10 for *w_s_*, 0 to 10 for *w_n_*, 25 to 200 for *w_d_*, and 0.5 to 15 for *d*.

### Adjusting Certain Parameters Used in the Rule Scoring and the Item Scoring Functions

Both the rule scoring and item scoring functions have several parameters. On the basis of the preferences of the users of the automated explanation function and the specific needs of the particular health care application, the developer of the automated explanation function could change some of these parameters from their default values. In the UWM test case used in this study, all association rules used by our automated explanation method were actionable. For some other predictive modeling problems, certain rules used by our automated explanation method are nonactionable [36]. In this case, if we want to allow some nonactionable rules to rank higher than some non-top-scored actionable rules on any patient, we need to reduce the weight *w_a_*. Similarly, if we want to allow some nonactionable items to rank higher than some actionable items in any non-top-scored rule that our automated explanation method finds for any patient, we need to reduce the weight *w_b_*.

### Considerations on the Threshold That Is Used to Determine the Top Rules That Will Be Displayed by Default

Different patients have different distributions of the ranking scores for the association rules found for the patients. No single threshold on the ranking score works for all patients. Thus, we use a threshold on the number of rules rather than a threshold on the ranking score to determine the top rules that will be displayed by default. This is similar to the case with a web search engine such as Google. Google does not use any ranking score threshold to determine the search results that will be displayed on each search result page. Instead, by default, Google displays 10 search results on each search result page. The user can request to see more search results by clicking the *next* button.

### Considerations Regarding Potential Clinical Use

Understanding how a predictive model works requires a global interpretation. Understanding a single prediction of a model requires only local interpretation [29,30]. Our automated explanation method provides local interpretations. For clinical applications, the user of the automated explanation function is frequently a clinician who has little or no background in machine learning, can see only the prediction results but not the internal of the machine learning predictive model, cares about understanding the prediction on an individual patient but not much about how the predictive model works internally, and possibly does not even know which predictive model is used because the model is often embedded in the clinical software. In this case, it does not matter whether the explanations provided by the automated explanation function match how the predictive model works internally, as long as the explanations can help the user understand the prediction for a specific patient. For a patient predicted to have a poor outcome, our automated explanation method will give the same set of explanations regardless of which machine learning model is used to make the prediction. In the case where a deep learning model built on longitudinal data is used to make predictions, we can use the method proposed in our paper [45] to extract temporal features from the deep learning model and longitudinal data, use these temporal features to convert longitudinal data to tabular data, and then apply our automated explanation method to a predictive model built on the tabular data.

To use our automated explanation method in clinical practice, we could implement our automated explanation method together with our explanation ranking method as a software library with an application programming interface. For any clinical decision support software that uses a machine learning predictive model, we could use the application programming interface to add the automated explanation function into the software to explain the model’s predictions.

### Related Work

As surveyed in the book written by Molnar [29] and the previous papers written by several research groups [30,46-48], other researchers have proposed many automated methods to explain machine learning predictions. Some of these methods are used for traditional machine learning algorithms, whereas others are specifically designed for deep learning algorithms [48]. The explanations given by most of these methods are not in a rule form. Many of these methods can handle only a specific machine learning algorithm or degrade the performance measures of the predictive model. None of these methods can automatically suggest tailored interventions. Ribeiro et al [49] and Rudin and Shaposhnik [50] used rules to explain any machine learning model’s predictions automatically. However, automatically recommending tailored interventions is still beyond the reach of the methods proposed by Ribeiro et al [49] and Rudin and Shaposhnik [50], as the rules are not generated until the prediction time. In comparison, our automated explanation method mines the association rules before the prediction time, provides rule-based explanations, works for any machine learning predictive model built on tabular data, does not degrade model performance, and automatically recommends tailored interventions. Compared with other types of explanations, rule-based explanations can more directly recommend tailored interventions and are easier to understand.

As surveyed in previous studies [39,51,52], association rules have been used in various applications to discover interesting patterns in the data and to make predictions. Various methods have been proposed to rank the rules mined from a data set for these purposes [39,51-55]. In comparison, we mine and rank association rules to automatically explain machine learning predictions and to recommend tailored interventions.

### Limitations

This work has three limitations that are excellent areas for future work:

1. This study used data from a single health care system. In the future, it would be beneficial to test our explanation ranking method on data from other health care systems.
2. This study tested our explanation ranking method for predicting one specific target in one disease. In the future, it would be beneficial to test our method on predictive modeling problems that address other prediction targets and diseases.
3. The data set used in this work contains no information on patients’ encounters outside the UWM. This forced us to limit the prediction target to asthma hospital encounters at the UWM rather than asthma hospital encounters in any health care system. In addition, the features used in this study were computed solely from the data recorded for the patients’ encounters at the UWM. In the future, it would be worth investigating how the top few explanations produced by our explanation ranking method would differ if we have data on the patients’ encounters in other health care systems.

### Conclusions

In this study, we developed a method to rank the rule-based explanations generated by our automated explanation method for machine learning predictions. Within a negligible amount of time, our explanation ranking method ranks the explanations and returns the few highest-ranked explanations. These few explanations typically have high quality and low redundancy. Many of them provide useful insights on the various aspects of the patient’s situation, which cannot be easily obtained by viewing the patient’s data in the current EHR system. Both our automated explanation method and our explanation ranking method are designed based on general computer science principles and rely on no special property of any specific disease, prediction target, patient cohort, or health care system. Although only tested in the case of predicting asthma hospital encounters in patients with asthma, our explanation ranking method is general and can be used for any predictive modeling problem on any tabular data set. The explanation ranking module is an essential component of the automated explanation function, which addresses the interpretability issue that deters the widespread adoption of machine learning predictive models in clinical practice. In the next few years, we plan to test our explanation ranking method on predictive modeling problems addressing other diseases as well as on data from other health care systems.

## Appendix

Multimedia Appendix 1 A summary of the demographic and clinical characteristics of patients with asthma at the University of Washington Medicine.

## Abbreviations

ED: emergency department
EHR: electronic health record
ICD: International Classification of Diseases
UWM: University of Washington Medicine
XGBoost: extreme gradient boosting
