# Supplementary material for: Ranking Rule-Based Automatic Explanations for Machine Learning Predictions on Asthma Hospital Encounters in Patients With Asthma: Retrospective Cohort Study
Source: JMIR Med Inform. 2021 Aug 11;9(8):e28287. doi: 10.2196/28287 (PMC8387888; doi:10.2196/28287)
Supplement: Multimedia Appendix 1 [file medinform_v9i8e28287_app1.pdf]

Appendix

Table 1. A summary of the demographic and clinical characteristics of the UWM asthma patients during 2011-2017.

Characteristic	Data instances linked to no asthma hospital encounter in the succeeding 12 months (*N*=67,060), *n* (%)	Data instances linked to asthma hospital encounters in the succeeding 12 months (*N*=1,184), *n* (%)	Data instances (*N*=68,244), *n* (%)
**Age**			
<40	22,993 (34.29)	466 (39.36)	23,459 (34.38)
40 to 65	33,306 (49.67)	583 (49.24)	33,889 (49.66)
65+	10,761 (16.05)	135 (11.40)	10,896 (15.97)
**Gender**			
Male	23,647 (35.26)	551 (46.54)	24,198 (35.46)
Female	43,413 (64.74)	633 (53.46)	44,046 (64.54)
**Race**			
White	47,240 (70.44)	507 (42.82)	47,747 (69.97)
Black or African American	7,900 (11.78)	520 (43.92)	8,420 (12.34)
Asian	5,625 (8.39)	96 (8.11)	5,721 (8.38)
American Indian or Alaska native	1,326 (1.98)	32 (2.70)	1,358 (1.99)
Native Hawaiian or other Pacific islander	659 (0.98)	14 (1.18)	673 (0.99)
Unknown or not reported	4,310 (6.43)	15 (1.27)	4,325 (6.34)
**Ethnicity**			
Non-Hispanic	55,247 (82.38)	1,062 (89.70)	56,309 (82.51)
Hispanic	3,444 (5.14)	82 (6.93)	3,526 (5.17)
Unknown or not reported	8,369 (12.48)	40 (3.38)	8,409 (12.32)
**Insurance**			
Private	39,585 (59.03)	424 (35.81)	40,009 (58.63)
Public	28,031 (41.80)	756 (63.85)	28,787 (42.18)
Self-paid or charity	1,301 (1.94)	65 (5.49)	1,366 (2.00)
**Number of years since the earliest asthma-related encounter in the data set**			
≤3	59,887 (89.30)	986 (83.28)	60,873 (89.20)
>3	7,173 (10.70)	198 (16.72)	7,371 (10.80)
**Asthma medication prescription**			
Short-acting inhaled beta-2 agonist	46,798 (69.79)	1,010 (85.30)	47,808 (70.05)
Inhaled corticosteroid	28,263 (42.15)	626 (52.88)	28,889 (42.33)
Long-acting beta-2 agonist and inhaled corticosteroid combination	21,516 (32.08)	499 (42.15)	22,015 (32.26)
Systemic corticosteroid	18,085 (26.97)	614 (51.86)	18,699 (27.40)
Long-acting beta-2 agonist	11,919 (17.77)	374 (31.59)	12,293 (18.01)
Leukotriene modifier	7,970 (11.88)	201 (16.98)	8,171 (11.97)
Mast cell stabilizer	43 (0.06)	4 (0.34)	47 (0.07)
**Comorbidity**			
Anxiety or depression	19,513 (29.10)	372 (31.42)	19,885 (29.14)
Gastroesophageal reflux	12,053 (17.97)	238 (20.10)	12,291 (18.01)
Allergic rhinitis	11,277 (16.82)	172 (14.53)	11,449 (16.78)
Obesity	7,668 (11.43)	177 (14.95)	7,845 (11.50)
Sinusitis	7,172 (10.69)	89 (7.52)	7,261 (10.64)
Sleep apnea	4,468 (6.66)	88 (7.43)	4,556 (6.68)
Eczema	3,825 (5.70)	66 (5.57)	3,891 (5.70)
Chronic obstructive pulmonary disease	3,693 (5.51)	133 (11.23)	3,826 (5.61)
Cystic fibrosis	60 (0.09)	1 (0.08)	61 (0.09)
Premature birth	8 (0.01)	0 (0.00)	8 (0.01)

Bronchopulmonary dysplasia	1 (0.00)	0 (0.00)	1 (0.00)
**Smoking status**			
Former smoker	15,309 (22.83)	221 (18.67)	15,530 (22.76)
Current smoker	13,826 (20.62)	255 (21.54)	14,081 (20.63)
Never smoker or unknown	37,925 (56.55)	708 (59.80)	38,633 (56.61)

Table 2. A summary of the demographic and clinical characteristics of the UWM asthma patients in 2018.

Characteristic	Data instances linked to no asthma hospital encounter in the succeeding 12 months (*N*=14,426), *n* (%)	Data instances linked to asthma hospital encounters in the succeeding 12 months (*N*=218), *n* (%)	Data instances (*N*=14,644), *n* (%)
**Age**			
<40	4,746 (32.90)	77 (35.32)	4,823 (32.94)
40 to 65	6,683 (46.33)	111 (50.92)	6,794 (46.39)
65+	2,997 (20.78)	30 (13.76)	3,027 (20.67)
**Gender**			
Male	5,138 (35.62)	100 (45.87)	5,238 (35.77)
Female	9,288 (64.38)	118 (54.13)	9,406 (64.23)
**Race**			
White	10,103 (70.03)	110 (50.46)	10,213 (69.74)
Black or African American	1,491 (10.34)	79 (36.24)	1,570 (10.72)
Asian	1,307 (9.06)	18 (8.26)	1,325 (9.05)
American Indian or Alaska native	273 (1.89)	8 (3.67)	281 (1.92)
Native Hawaiian or other Pacific islander	129 (0.89)	2 (0.92)	131 (0.89)
Unknown or not reported	1,123 (7.78)	1 (0.46)	1,124 (7.68)
**Ethnicity**			
Non-Hispanic	12,370 (85.75)	196 (89.91)	12,566 (85.81)
Hispanic	830 (5.75)	20 (9.17)	850 (5.80)
Unknown or not reported	1,226 (8.50)	2 (0.92)	1,228 (8.39)
**Insurance**			
Private	10,692 (74.12)	108 (49.54)	10,800 (73.75)
Public	7,841 (54.35)	182 (83.49)	8,023 (54.79)
Self-paid or charity	459 (3.18)	25 (11.47)	484 (3.31)
**Number of years since the earliest asthma-related encounter in the data set**			
≤3	10,442 (72.38)	124 (56.88)	10,566 (72.15)
>3	3,984 (27.62)	94 (43.12)	4,078 (27.85)
**Asthma medication prescription**			
Short-acting inhaled beta-2 agonist	9,540 (66.13)	164 (75.23)	9,704 (66.27)
Inhaled corticosteroid	6,069 (42.07)	108 (49.54)	6,177 (42.18)
Long-acting beta-2 agonist and inhaled corticosteroid combination	4,425 (30.67)	83 (38.07)	4,508 (30.78)
Systemic corticosteroid	4,043 (28.03)	120 (55.05)	4,163 (28.43)
Long-acting beta-2 agonist	2,456 (17.02)	62 (28.44)	2,518 (17.19)
Leukotriene modifier	2,130 (14.77)	46 (21.10)	2,176 (14.86)
Mast cell stabilizer	13 (0.09)	1 (0.46)	14 (0.10)
**Comorbidity**			
Anxiety or depression	4,284 (29.70)	62 (28.44)	4,346 (29.68)
Gastroesophageal reflux	2,611 (18.10)	46 (21.10)	2,657 (18.14)
Allergic rhinitis	2,069 (14.34)	26 (11.93)	2,095 (14.31)
Obesity	1,579 (10.95)	25 (11.47)	1,604 (10.95)
Sinusitis	1,357 (9.41)	15 (6.88)	1,372 (9.37)
Sleep apnea	1,475 (10.22)	24 (11.01)	1,499 (10.24)

Eczema	732 (5.07)	11 (5.05)	743 (5.07)
Chronic obstructive pulmonary disease	902 (6.25)	30 (13.76)	932 (6.36)
Cystic fibrosis	17 (0.12)	0 (0.00)	17 (0.12)
Bronchopulmonary dysplasia	4 (0.03)	0 (0.00)	4 (0.03)
Premature birth	2 (0.01)	0 (0.00)	2 (0.01)
**Smoking status**			
Former smoker	3,453 (23.94)	41 (18.81)	3,494 (23.86)
Current smoker	3,193 (22.13)	49 (22.48)	3,242 (22.14)
Never smoker or unknown	7,780 (53.93)	128 (58.72)	7,908 (54.00)
